# Clinical outcomes and prognostic factors of extended calcium hydroxide medication followed by a bioceramic apical barrier in immature permanent teeth: a retrospective study

**DOI:** 10.1186/s12903-026-09188-4

**Published:** 2026-07-11

**Authors:** Ke Xu, Xuening Gu, Xiaoyin Xu, Suqian Shen, Le Lu

**Affiliations:** Department of Endodontics, Suzhou Stomatological Hospital, No.1366, Suzhan Road, Suzhou, China

**Keywords:** Calcium hydroxide, Apical barrier, Immature permanent teeth, Periapical healing

## Abstract

Managing non-vital immature permanent teeth with extensive periapical lesions remains a clinical challenge. This retrospective cohort study aimed to evaluate the clinical and radiographic outcomes of a staged protocol utilizing extended calcium hydroxide medication followed by a bioceramic apical barrier, and to identify prognostic factors influencing strict radiographic healing.

**Methods **A total of 124 immature permanent teeth with necrotic pulps and open apices, treated at a single center between January 2021 and December 2024, were included. The protocol mandated intracanal calcium hydroxide dressing for a minimum of 1 month, periodically replaced until absolute canal dryness was achieved, followed by the placement of an iRoot BP Plus apical barrier. Periapical lesion areas were quantitatively measured on calibrated radiographs using ImageJ software. Outcomes were classified as strict success (asymptomatic with complete radiographic resolution), improved (asymptomatic and functional with a quantifiable reduction in radiolucency), or failed. Multivariate logistic regression was performed to identify independent predictors of non-strict success.

**Results** Over a median follow-up of 12.0 months (interquartile range [IQR]: 8.0–25.0), the overall success rate (strict success + improved) was 96.8% (120/124), while the strict success rate was 83.1% (103/124). Mean periapical lesion area decreased significantly from 50.83 ± 47.71 mm² preoperatively to 4.93 ± 25.79 mm² at the final follow-up (*P* < 0.001). Multivariate analysis identified four independent predictors significantly associated with incomplete radiographic healing: anterior tooth location (Odds Ratio [OR] = 4.21, 95% CI: 1.38–12.75; *P* = 0.012), initial lesion area ≥ 50 mm² (OR = 3.87, 95% CI: 1.26–11.81; *P* = 0.018), apical foramen diameter ≥ 1 mm (OR = 3.54, 95% CI: 1.19–10.56; *P* = 0.024), and the presence of a sinus tract (OR = 3.96, 95% CI: 1.27–12.37; *P* = 0.018).

**Conclusions** A staged protocol utilizing extended calcium hydroxide followed by an iRoot BP Plus apical barrier yields highly predictable outcomes for non-vital immature permanent teeth. While overall functional retention is highly successful, anterior tooth location, large initial lesions, wide apical foramina, and sinus tracts independently delay complete radiographic resolution.

## Introduction

Treating necrotic immature permanent teeth remains clinically challenging because interrupted root development creates thin dentinal walls, short roots, an unfavorable crown-to-root ratio, and wide open apices, complicating necrotic tissue removal, mechanical debridement, irrigation control, apical disinfection, and obturation [1]. Regenerative endodontic procedures (REPs) may allow further root maturation, but success depends on viable apical tissues, stem cell recruitment, adequate disinfection, and favorable case selection [1, 2]. Immature teeth with Cvek stage II–IV root development feature incomplete root formation, divergent or parallel canal walls, and open apices [3]. The recent European Society of Endodontology (ESE) S3-level guideline emphasizes that apexification remains a predictable and evidence-supported treatment for necrotic immature permanent teeth, particularly when regenerative potential is compromised by chronic infection, apical tissue damage, or patient-related factors [4]. In the present cohort, an apical barrier technique was preferred to provide a predictable and immediate seal for severely infected teeth with large lesions.

Bioceramic apical barriers, such as iRoot BP Plus, allow the immediate creation of an artificial apical stop, reducing the prolonged treatment time associated with traditional calcium hydroxide apexification. However, immediate barrier placement in teeth with wide open apices, large periapical lesions, and weeping apices (persistent exudation) may compromise material adaptation and increase extrusion risk. Therefore, a staged protocol using intracanal calcium hydroxide medication before definitive bioceramic barrier placement is deliberately chosen to improve disinfection and establish a completely dry environment suitable for controlled apical plug formation [5–7].

Calcium hydroxide has long been used in apexification due to its antimicrobial activity and ability to induce hard tissue formation [8]. Nevertheless, prolonged exposure to calcium hydroxide may weaken immature roots; specifically, its high alkalinity denatures dentinal collagen fibrils, thereby reducing fracture resistance [9, 10]. To balance these benefits against potential risks, the protocol dictates that medication duration is strictly guided by clinical resolution— specifically, achieving an asymptoms completely dry canal (requiring ≥ 1 month)—rather than an arbitrarily prolonged timeframe. Recent evidence suggests this fracture risk may be effectively mitigated by conservative instrumentation to preserve radicular dentin, a timely transition to definitive obturation, and immediate intra-radicular bonded composite restorations to structurally reinforce the weakened tooth [11, 12].

Radiographic healing after endodontic treatment is traditionally assessed qualitatively using periapical indices [13]. However, quantitative image analysis provides an objective method for measuring lesion area and apical morphology. Although cone-beam computed tomography (CBCT) offers greater three-dimensional accuracy [14], routine CBCT follow-up is not always justified under radiation safety principles, and two-dimensional radiographs remain widely used in retrospective studies [15]. ImageJ-based calibrated measurements can therefore provide reliable quantitative data for evaluating healing dynamics and prognostic factors.

The aims of this retrospective cohort study were:

1. to evaluate the clinical and radiographic outcomes of a staged protocol consisting of extended calcium hydroxide intracanal medication (defined operationally as a mandatory minimum of 1 month, dynamically extending to ≥ 3 months in severe cases until absolute canal dryness is achieved) followed by an iRoot BP Plus apical barrier in necrotic immature permanent teeth; and
2. to identify prognostic factors associated with failure to achieve complete radiographic healing.

It was hypothesized that this staged protocol would yield a high overall success rate, while baseline disease severity, including large periapical lesions, wide apical foramina, and sinus tracts——would negatively influence strict radiographic healing.

## Materials and methods

### Study design and ethics

This retrospective cohort study was designed and reported in accordance with the STROBE guidelines. The study protocol was approved by the Ethics Committee of Suzhou Stomatological Hospital, China (Approval No. SZKQYY-2021-H002). Due to the retrospective nature of the study, the requirement for informed consent was waived. All patient data were anonymized before analysis.

Dental records from January 2021 to December 2024 were screened using a consecutive sampling method. To avoid clustering effects, only one tooth per patient was included. To handle missing data, a complete case analysis approach was adopted; patients with missing or incomplete clinical or radiographic records were strictly excluded prior to analysis to avoid imputation bias. Selection bias was mitigated by rigorously applying predefined inclusion and exclusion criteria, while detection bias was minimized by utilizing two independent evaluators blinded to treatment outcomes during radiographic measurements.

As a retrospective study, the sample size was determined by the number of eligible records available during the study period. A post-hoc power analysis was conducted based on an anticipated overall success rate of 80%, an alpha error probability of 0.05, and an expected odds ratio of 3.0 for primary clinical predictors; the final sample of 124 teeth provided > 80% statistical power for the multivariate logistic regression.

### Participants

Inclusion criteria were as follows: (1) patients aged 11–40 years; (2) presence of a non-vital immature permanent tooth with Cvek stage III–IV root development [3]; (3) measurable baseline periapical radiolucency associated with disruption of the lamina dura and/or widening of the periodontal ligament space; (4) treatment using a staged protocol consisting of intracanal calcium hydroxide medication for ≥ 1 month followed by bioceramic apical barrier placement; (5) complete baseline and follow-up radiographic records; and (6) minimum follow-up period of 6 months.

Exclusion criteria were as follows: (1) concomitant root fracture or advanced periodontal disease; (2) systemic diseases known to affect healing; and (3) radiographs with severe distortion, marked elongation or foreshortening, or unclear lesion margins unsuitable for quantitative analysis.

### Clinical treatment protocol

All procedures for this combined protocol (integrating classical calcium hydroxide disinfection with an artificial bioceramic plug) were performed by experienced endodontists (including the authors) under rubber dam isolation. After access cavity preparation, the working length was determined using an electronic apex locator and confirmed radiographically when necessary. Root canals were gently instrumented using nickel–titanium rotary files (Plex; Eurofile, China) with a conservative crown-down approach to minimize the weakening of thin dentinal walls.

During the inter-appointment phase, each canal was irrigated with approximately 20 mL of 2.5% sodium hypochlorite per session via a side-vented irrigation needle with gentle delivery to prevent extrusion. A premixed aqueous calcium hydroxide paste (ApexCal; Ivoclar Vivadent, Liechtenstein) was placed approximately 1 mm short of the radiographic apex using the manufacturer’s proprietary delivery tips and lentulo spirals for uniform adaptation. The access cavity was sealed with a 3-mm layer of glass ionomer cement (GC Fuji IX GP; GC, Japan). Calcium hydroxide medication was maintained for at least 1 month and replaced as needed until clinical signs of infection resolved, definded as absence of pain, swelling, sinus tract, and persistent intracanal exudation. Duration was determined by clinical response rather than a fixed schedule. Cases achieving canal dryness at the initial session were not excluded. However, definitive barrier placement was strictly delayed for a minimum of 1 month. This minimum duration was mandated not only to confirm a stable, dry environment, but primarily to maximize chemical disinfection via the alkaline action of calcium hydroxide, compensating for the limited mechanical instrumentation permitted in thin-walled immature roots. Once infection fully resolved, rather than waiting for natural calcified barrier formation, the procedure transitioned directly to artificial apical plug creation.

Before barrier placement, canals were irrigated with copious 2.5% sodium hypochlorite using passive ultrasonic activation to remove residual calcium hydroxide, then completely dried with sterile paper points until devoid of any exudation. A 3–5 mm apical barrier of iRoot BP Plus (Innovative Bioceramix, Canada) was placed and condensed with hand pluggers following the protocol described by Tolibah et al. [16]. After radiographic verification of barrier position and thickness, the remaining canal space was obturated with thermoplasticized gutta-percha (B&L Biotech, South Korea) and a bioceramic sealer (iRoot SP; Innovative Bioceramix, Canada). All teeth received bonded composite resin restorations within two weeks of obturation to provide coronal seal and structural reinforcement; immediate cuspal coverage was not routinely performed due to the conservative nature of the access cavities.

Medication overextension and apical barrier overfill were recorded and included in the statistical analysis rather than excluded, accurately reflecting real-world clinical practice and avoiding selection bias.

### Radiographic acquisition and quantitative assessment

Retrospective periapical radiographs (DICOM format) were obtained using either the paralleling technique (with Rinn XCP positioning holders to aid angulation control) or the bisecting-angle technique, depending on pediatric patient cooperation. Because consistent angulation and uniform device settings could not be guaranteed retrospectively, severely distorted radiographs were excluded to minimize projection errors. To mathematically handle retrospective magnification variances and improve measurement validity, each radiograph was individually calibrated using its DICOM-embedded pixel spacing via the “Set Scale” function in ImageJ (NIH, USA). Continuous 2D area measurement was deliberately chosen over ordinal Periapical Index (PAI) scoring to provide greater quantitative sensitivity. Routine CBCT was precluded by ALARA radiation safety principles.

A periapical lesion was defined as a measurable radiolucency disrupting the lamina dura or periodontal ligament space. Lesions were manually traced using the polygon tool (rather than automated thresholding). For unclear borders, the most conservative identifiable margin was traced to avoid overestimating healing. Baseline apical foramen diameter was measured as the maximum apical opening width; specifically, using the straight-line tool in ImageJ, the maximum linear distance between the innermost dentinal walls at the most apical extent of the root was recorded.

Image enhancements were restricted to brightness/contrast adjustments. Two independent, calibrated endodontists, strictly blinded to chronological timepoints and outcomes, evaluated all radiographs; disagreements were resolved by consensus. Additionally, baseline demographic data, tooth location (anterior vs. posterior), and the precise etiology of pulp necrosis (dens evaginatus, trauma, or caries) were systematically extracted from dental charts for subsequent prognostic analysis.

### Outcome assessment

Treatment outcomes at the final follow-up were classified into three mutually exclusive groups, modified from established endodontic healing criteria [17]:

1. Strict success (healed): absence of clinical symptoms (negative percussion /palpation, normal mobility, no sinus tract) and complete radiographic resolution (normal trabecular pattern and uniform periodontal ligament space).
2. Improved (incomplete healing): completely asymptomatic and functional, with a quantifiable reduction in radiolucency compared with baseline.
3. Failure: persistent/new clinical symptoms, unchanged/increased radiolucency, or need for further intervention.

For the regression analysis, the dependent outcome was dichotomized as strict success versus non-strict success (improved + failed cases).

### Statistical analysis

Analyses were performed using SPSS 26.0 (IBM Corp., USA). Variables were summarized using standard descriptive statistics and compared via Chi-square, Fisher’s exact, or appropriate non-parametric continuous tests.

To reduce model instability, continuous predictors were dichotomized using clinical thresholds: baseline lesion area (≥ 50 mm² vs. <50 mm² for extensive lesions) and apical foramen diameter (≥ 1 mm vs. <1 mm). Predictor selection for the multivariate model was based on biological plausibility and statistical significance; variables with *P* < 0.10 in the univariate analysis, alongside clinically relevant predictors, were entered into a multivariate logistic regression to identify independent predictors of non-strict success. Model fit was assessed using the Hosmer-Lemeshow test, and multicollinearity was verified using the variance inflation factor (VIF < 5).

Because precise time-to-healing dates were not consistently available retrospectively, Cox proportional hazards analysis could not be performed. Instead, a sensitivity analysis stratified by follow-up duration was conducted to account for the time-dependency of healing. Statistical significance was set at a two-sided *P* < 0.05.

## Results

### Baseline characteristics and treatment outcomes

A total of 124 teeth (124 patients; mean age 19.8 ± 6.4 years) were included. Detailed baseline demographic, clinical, and radiographic characteristics are summarized in Table 1. Inter-examiner reliability for radiographic measurements was excellent (ICC = 0.94). Notably, dens evaginatus was the predominant etiology (62.9%), and 40.3% of cases required ≥ 3 months of calcium hydroxide medication to achieve clinical dryness. Medication or barrier overextension did not significantly affect treatment outcomes (*P* > 0.05).

**Table 1:** Demographic characteristics, clinical variables, and univariate analysis of factors associated with treatment outcomes under strict and loose success criteria

Demographic variables	Category	*N* (%)	Healed (*n*, %)	Healing (*n*, %)	Failure (*n*, %)	*P*	Success strict (*n*%)	*P*	Success loose (*n*%)	*P*
Sex	Male	42 (33.9%)	40 (95.2%)	2 (4.8%)	0 (0%)	0.183	95.2%	0.152	100%	0.102
	Female	82 (66.1%)	63 (76.8%)	15 (18.3%)	4 (4.9%)		76.8%		95.1%	
Age (y)	≤ 18	58 (46.8%)	50 (86.2%)	8 (13.8%)	0 (0%)	0.052	86.2%	**0.045**	100%	**0.032**
	> 18	66 (53.2%)	53 (80.3%)	9 (13.6%)	4 (6.1%)		80.3%		93.9%	
Tooth variables										
Tooth type	Anterior	32 (25.8%)	20 (62.5%)	8 (25.0%)	4 (12.5%)	**0.01**	62.5%	**0.01**	87.5%	**0.024**
	Premolar	86 (69.4%)	77 (89.5%)	9 (10.5%)	0 (0%)		89.5%		100%	
	Molar	6 (4.8%)	6 (100%)	0 (0%)	0 (0%)		100%		100%	
Size of lesion area	< 20 mm²	30 (24.2%)	28 (93.3%)	2 (6.7%)	0 (0%)	**0.032**	93.3%	**0.032**	100%	**0.025**
	≥ 20, < 50 mm²	46 (37.1%)	35 (76.1%)	11 (23.9%)	0 (0%)		76.1%		100%	
	≥ 50 mm²	48 (38.7%)	37 (77.1)	7 (14.6%)	4 (8.3%)		77.1%		91.7%	
Size of apical foramen	< 1 mm	58 (46.8%)	52 (89.7%)	6 (10.3%)	0 (0%)	0.039	89.7%	**0.039**	100%	**0.028**
	≥ 1 mm	66 (53.2%)	51 (77.3%)	11 (16.7%)	4 (6.0%)		77.3%		94.0%	
Etiology of pulp necrosis	Caries	24 (19.4%)	20 (83.3%)	3 (12.5%)	1 (4.2%)	0.088	83.3%	0.088	95.8%	0.075
	Dens evaginatus	78 (62.9%)	63 (80.8%)	11 (14.1%)	4 (5.1%)		80.8%		94.9%	
	Trauma	22 (17.7%)	20 (90.9%)	2 (9.1%)	0 (0%)		90.9%		100%	
Sinus tract	Presence	40 (32.3%)	30 (75.0%)	6 (15.0%)	4 (10.0%)	**0.021**	75.0%	**0.021**	90.0%	**0.018**
	Absence	84 (67.7%)	73 (86.9%)	11 (13.1%)	0 (0%)		86.9%		100%	
Tooth Mobility	Presence	10 (8.1%)	8 (80.0%)	1 (10.0%)	1 (10.0%)	0.064	80.0%	0.064	90.0%	0.052
	Absence	114 (91.9%)	95 (83.3%)	16 (14.0%)	3 (2.6%)		83.3%		97.4%	
Treatment variables										
Medication duration(m)	1–3	74 (59.7%)	60 (81.1%)	11 (14.9%)	3 (4.0%)	0.291	81.18%	0.276	95.9%	0.248
	≥ 3	50 (40.3%)	40 (80.0%)	9 (18.0%)	1 (2.0%)		80.0%		98.0%	
Medication quality	Underfill	26 (21.0%)	20 (76.9%)	4 (15.4%)	2 (7.7%)	0.212	76.9%	0.212	92.3%	0.192
	Adequate	76 (61.3%)	63 (82.9%)	11 (14.5%)	2 (2.6%)		82.9%		97.4%	
	Overfill	22 (17.7%)	17 (77.3%)	5 (22.7%)	0 (0%)		77.3%		100.0%	
Apical barrier quality	Underfill	16 (12.9%)	11 (68.8%)	3 (18.7%)	2 (12.5%)	0.186	68.8%	0.186	87.5%	0.162
	Adequate	100 (80.6%)	83 (83.0%)	15 (15.0%)	2 (2.0%)		83.0%		98.0%	
	Overfill	8 (6.5%)	6 (75.0%)	2 (25.0%)	0 (0%)		75.0%		100.0%	

Over a median follow-up of 12.0 months (IQR: 8.0–25.0;range: 6.0–77.0 months), the overall success rate (strict success + improved) was 96.8% (120/124). Strict success (complete radiographic healing) was achieved in 83.1% (103/124) of cases. Four teeth (3.2%) failed; notably, all failures occurred in anterior teeth, whereas premolars and molars achieved 100% overall success. No root fractures were recorded. Representative radiographic healing patterns are illustrated in Fig. 1.

**Fig. 1:**
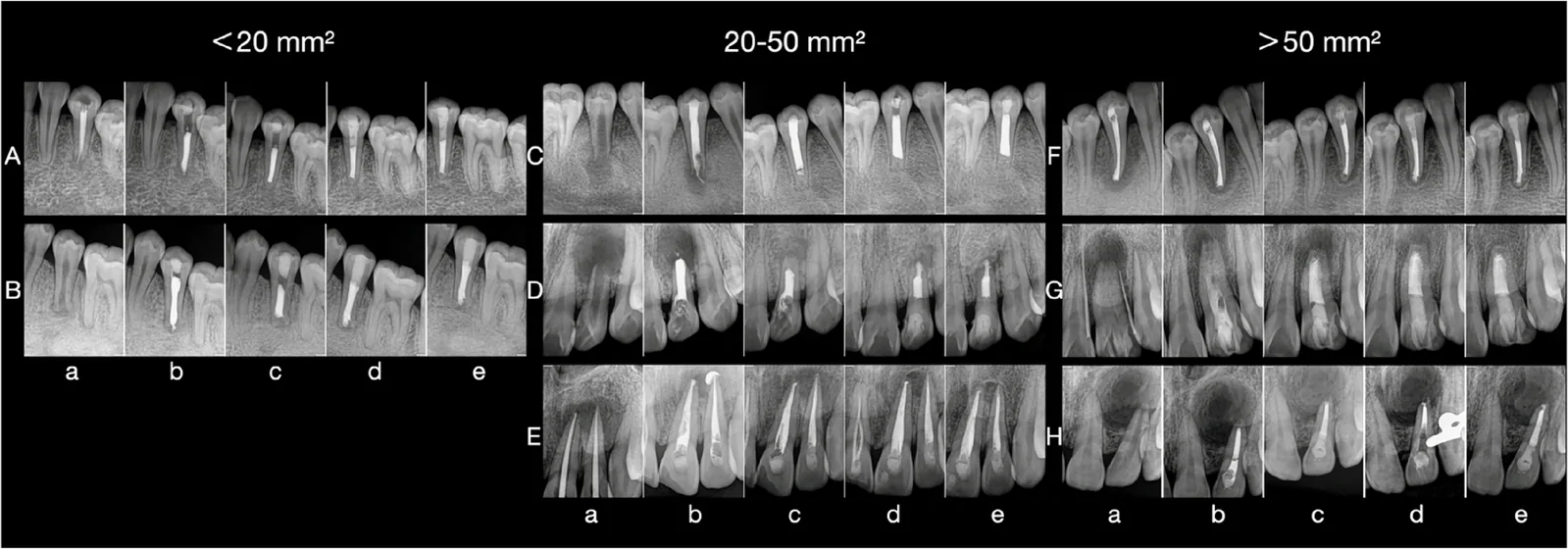
Representative radiographic outcomes stratified by preoperative lesion size. Columns **a**–**e** indicate the treatment timeline: **a** preoperative radiograph; **b** immediate post-calcium hydroxide dressing; **c** follow-up during medication; **d** immediate post-apical barrier placement; and **e** final follow-up. Rows **A**–**H** denote individual representative cases categorized by baseline lesion area. Left panel (< 20 mm²): **A** Healed case; **B** Improved case. Middle panel (20–50 mm²): **C** Healed case; **D** Improved case; **E** Failed case. Right panel (> 50 mm²): **F** Healed case; **G** Improved case; **H** Failed case

### Univariate analysis of prognostic factors

As detailed in Table 1, univariate analysis identified that anterior tooth location, baseline lesions ≥ 50 mm², apical foramen diameter ≥ 1 mm, presence of a sinus tract, and age > 18 years were significantly associated with lower strict success rates (*P* < 0.05). Conversely, pulp necrosis etiology (*P* = 0.088), medication duration, and technical quality parameters (medication/barrier adaptation) were not significant predictors.

### Multivariate logistic regression analysis

After adjusting for confounders, the multivariate model identified four independent predictors associated with incomplete radiographic (non-strict) success (Table 2): anterior tooth location, lesion area > 50 mm², apical foramen diameter ≥ 1 mm, and presence of a sinus tract.

**Table 2:** Multivariate logistic regression model identifying independent predictors of incomplete radiographic healing (non-strict success). Multiple logistic regression for the association of the independent variables and treatment outcomes under strict criteria

Predictor Variables	Adjusted OR (95% CI)	*P* value
Demographic factors		
Sex(Male vs. Female [Ref])	1.08 (0.38–3.11)	0.892
Age (y) (> 18 years vs. ≤18 years [Ref])	1.62 (0.52–5.04)	0.401
Baseline clinical factors		
Tooth type (Anterior vs. Posterior [Ref])	4.21 (1.38–12.75)	**0.012**
Initial lesion area(≥ 50 mm² vs. <50 mm² [Ref])	3.87 (1.26–11.81)	**0.018**
Apical foramen diameter (≥ 1 mm vs. <1 mm [Ref])	3.54 (1.19–10.56)	**0.024**
Sinus tract (Present vs. Absent [Ref])	3.96 (1.27–12.37)	**0.018**
Tooth mobility (Present vs. Absent [Ref])	1.45 (0.39–12.37)	0.574
Etiology (Trauma/Caries vs. Dens evaginatus [Ref])*	0.82 (0.21–3.20)	0.778
Treatment-related factors		
Medication duration (≥ 3 months vs. 1–3 months [Ref])	0.92 (0.33–2.58)	0.877
Medication quality (Adequate vs. Over/Underfill [Ref])	0.89 (0.30–2.67)	0.841
Apical barrier quality (Adequate vs. Over/Underfill [Ref])	0.91 (0.31–2.70)	0.859

* *OR* Odds Ratio, *CI* Confidence Interval, *Ref* Reference category. Significant *P*-values (< 0.05) are highlighted in bold. For etiology, given the predominance of dens evaginatus, it was utilized as the baseline reference category

Age, pulp necrosis etiology, and all treatment-related variables lost statistical significance in the multivariate model. No significant multicollinearity was detected. Receiver operating characteristic (ROC) analysis demonstrated good discriminative ability for the model, yielding an area under the curve (AUC) of 0.861 (Fig. 2).

**Fig. 2:**
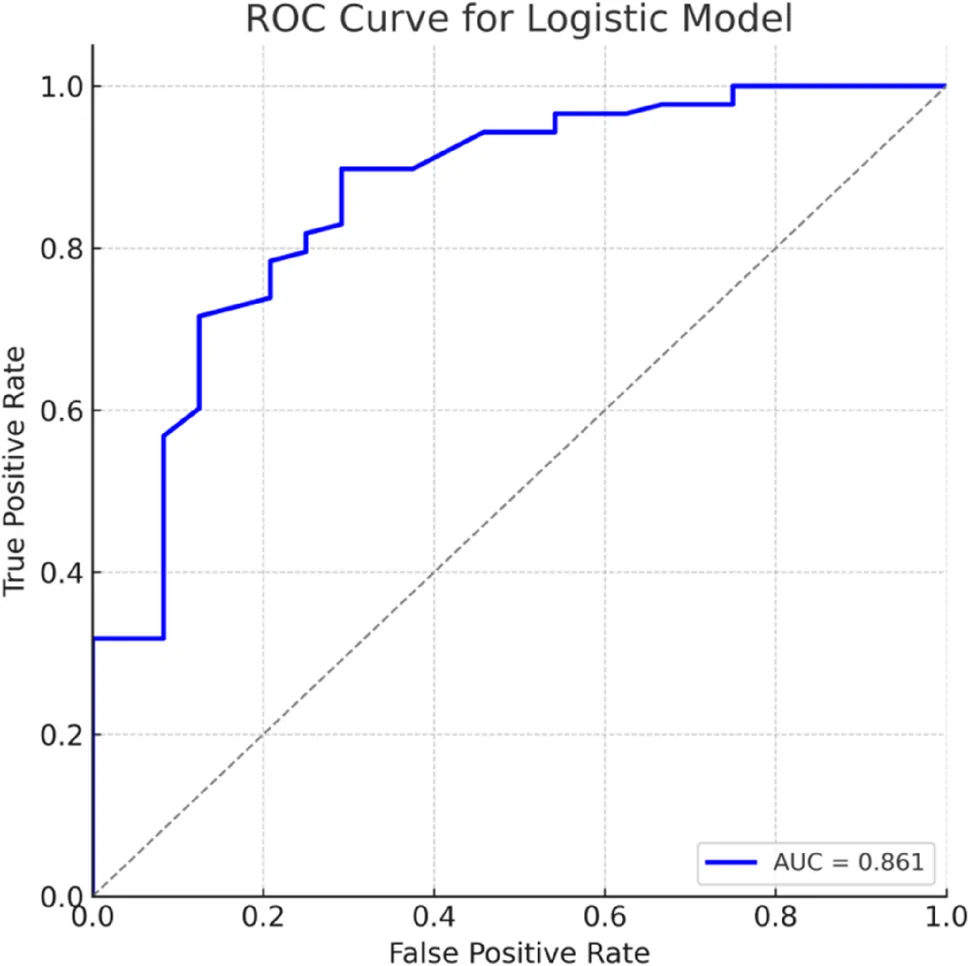
ROC curve for the multivariate logistic regression model. The receiver operating characteristic curve shows the discriminative ability of the multivariate model for predicting treatment outcome. The area under the curve was 0.861, indicating good predictive performance

## Discussion

The present study demonstrated that a staged protocol utilizing extended intracanal calcium hydroxide medication followed by a bioceramic apical barrier produced favorable outcomes in necrotic immature permanent teeth (96.8% overall success; 83.1% strict success).These findings support the acceptance of the primary hypothesis and closely align with current pediatric dentistry and endodontic clinical guidelines (such as the ESE S3-level guidelines), confirming that apexification remains a highly predictable treatment option when REPs are less feasible or less predictable [4–7, 18, 19]. However, it must be explicitly acknowledged that while the data strongly support the high functional retention, symptom resolution, and substantial radiographic reduction achieved, they cannot definitively prove comparative superiority over immediate apical barrier placement or REPs. Furthermore, given the retrospective, single-arm design lacking a control group or duration-response analysis, it cannot be definitively inferred that extended calcium hydroxide is strictly necessary for the observed outcomes.

The staged approach prioritizes infection control and moisture management prior to definitive sealing, which is crucial for wide open apices where persistent exudation may compromise apical plug adaptation. Although standard endodontic cases often resolve rapidly, the severe baseline infection in this cohort—characterized by massive lesions and weeping apices—necessitated extended calcium hydroxide dressing. This extended duration (often > 3 months) was not arbitrarily predetermined; rather, the medicament was periodically replaced, strictly guided by the cessation of exudation. Importantly, statistical analysis indicated that this prolonged medication duration did not significantly affect final outcomes, suggesting that achieving a completely dry, asymptomatic canal is biologically more critical than adhering to a predetermined chronological timeframe.

A major concern with extended calcium hydroxide apexification is the potential for cervical root fractures, as high alkalinity denatures dentinal collagen and reduces fracture resistance [9, 10]. Reassuringly, no cervical root fractures, recurrent infections, loss to follow-up, or restoration failures occurred. Root integrity and restoration status were rigorously assessed at each follow-up through clinical examination (periodontal probing, mobility testing, visual coronal seal inspection) and radiographic evaluation. This absence of complications is likely attributable to conservative instrumentation and immediate intra-radicular bonded composite restorations, which structurally reinforce the weakened roots [11, 12].

Multivariate analysis identified anterior tooth location, large lesion size, wide apical foramen, and sinus tract presence as independent risk factors for incomplete radiographic healing. Biologically, anterior tooth failures are closely tied to their trauma etiology; trauma-induced chronic lesions are highly susceptible to refractory extraradicular biofilms or true epithelial cysts [20]. Furthermore, extensive lesions frequently perforate the thin buccal cortical plate of anterior teeth, resulting in fibrous healing (an apical scar) that persists radiographically despite clinical success [21, 22].

Large baseline lesions (≥ 50 mm²) and sinus tracts represent a massive infection burden and the potential persistence of epithelial tracts. These conditions maintain direct communication between the root canal system and periradicular tissues, strongly resisting rapid nonsurgical disinfection and significantly delaying osseous remodeling [20, 22–24]. However, delayed radiographic resolution in these scenarios does not necessarily equate to clinical failure, as the vast majority of these teeth remained completely asymptomatic and functional.

A wide apical foramen (≥ 1 mm) emerged as another significant predictor. While a wide apex facilitates cell migration in REPs [25, 26], in apexification, it increases fluid communication with periapical tissues, complicates moisture control, and makes the controlled condensation and sealing of the bioceramic barrier technically demanding, thereby explaining its negative prognostic impact.

The technical quality and thickness of the bioceramic apical plug are important determinants of apexification outcomes. Here, a 3–5 mm iRoot BP Plus barrier was placed under dry conditions following the technique described by Tolibah et al. [16]. Although retrospective limitations precluded a statistically powered subgroup analysis of precise sub-millimeter plug thickness (e.g., 3 mm vs. 4–5 mm), recent evidence suggests that insufficient plug thickness (< 4 mm) may compromise treatment quality and increase failure risk [27]. The staged protocol used here allowed for optimal environmental control and moisture management prior to delivery, ensuring excellent material adaptation, which likely explain the favorable overall outcomes despite slight variations in barrier thickness.

Bioceramic delivery techniques and material bioactivity significantly impact clinical outcomes. Recent evidence shows that specific delivery methods influence material extrusion, postoperative pain, and procedure duration [28]; therefore, the staged, dry-canal protocol likely minimized extrusion risks and optimized material adaptation. Furthermore, bioceramics can induce apical calcified tissue barrier formation and continued root length development [29, 30]. Although the reliance on two-dimensional radiographs—combined with the extreme radiopacity of the bioceramic plug—precluded reliable evaluation of natural apical stop formation or subtle structural changes, the inherent osteogenic bioactivity of iRoot BP Plus undoubtedly contributed to the favorable healing observed.

Clinically, these findings suggest that large lesions, sinus tracts, and wide apices should not be regarded as contraindications for apexification, but rather as indicators potentially delayed radiographic healing. Patients with these features should receive appropriate counseling and longer follow-up. Finally, the inclusion of patients up to 40 years of age further suggests that successful healing after apexification is not limited to adolescents, provided that adequate disinfection and apical sealing are achieved.

## Limitations

Several limitations of the present study must be explicitly acknowledged. First, the retrospective, single-center design lacks a control group (e.g., immediate barrier placement or REPs), preventing definitive comparative conclusions regarding treatment superiority. Second, exclusive reliance on two-dimensional periapical radiographs introduces potential measurement bias; 2D imaging inherently masks the true volumetric extent of bone destruction and osseous healing, particularly in the buccolingual dimension. Third, due to considerable heterogeneity in follow-up duration, the minimum 6-month period is biologically insufficient to capture complete osseous regeneration for massive lesions or to reliably assess late cervical root fractures. Consequently, cases currently classified strictly as ‘improved’ may progress to ‘healed’ over an extended timeline, suggesting our reported strict success rate could be an underestimation. Fourth, the clinically dictated duration of calcium hydroxide medication introduced significant variability, complicating the determination of an optimal medication window. Finally, residual unmeasured confounders—specifically operator variability among multiple clinicians, differences in coronal restoration quality, occlusal loading dynamics, and patient compliance—could not be completely controlled and may have influenced the observed clinical outcomes.

## Conclusion

The staged protocol utilizing extended calcium hydroxide intracanal medication followed by iRoot BP Plus apical barrier placement was highly effective for necrotic immature permanent teeth, achieving a 96.8% overall success rate. Large periapical lesions, wide apical foramina, sinus tract presence, and anterior tooth location were associated with incomplete radiographic healing, though these factors did not preclude functional tooth retention. Given the study’s limitations, extended long-term follow-up and prospective comparative clinical trials are warranted to further validate these findings.

## Data Availability

The datasets generated and/or analyzed during the current study are not publicly avalable due to patient privacyrestrictions but are available from the corresponding author on reasonable request.
